# Patient and Care Team Perspectives on Social Determinants of Health Screening in Primary Care: A Qualitative Study

**DOI:** 10.1001/jamanetworkopen.2023.45444

**Published:** 2023-11-28

**Authors:** A. Caroline Rudisill, Meredith G.A. Eicken, Deeksha Gupta, Mark Macauda, Stella Self, Ann Blair Kennedy, Darin Thomas, Elise Kao, Mia Jeanty, Jackson Hartley

**Affiliations:** 1 Department of Health Promotion, Education, and Behavior, Arnold School of Public Health, University of South Carolina, Greenville; 2 Department of Medicine, Prisma Health, Upstate, University of South Carolina School of Medicine Greenville, Greenville; 3 Department of Epidemiology/Biostatistics, Arnold School of Public Health, University of South Carolina, Greenville; 4 Department of Biomedical Sciences, University of South Carolina School of Medicine Greenville, Greenville; 5 Addiction Medicine Center, Prisma Health, Greenville, South Carolina

## Abstract

**Importance:**

Health systems in the US are increasingly screening for social determinants of health (SDOH). However, guidance incorporating stakeholder feedback is limited.

**Objective:**

To examine patient and care team experiences in early implementation of SDOH screening in primary care.

**Design, Setting, and Participants:**

This qualitative study included cross-sectional analysis of SDOH screenings during primary care visits from February 22 to May 10, 2022, primary care team member interviews from July 6, 2022, to March 8, 2023, and patient stakeholder engagement on June 30, 2022. The setting was a large southeastern US health care system. Eligible patients were aged 18 years or older with completed visits in primary care.

**Exposure:**

Screening for SDOH in primary care.

**Main outcomes and Measures:**

Multivariable logistic regression evaluated patient (eg, age, race and ethnicity) and care team characteristics (eg, practice type), and screening completeness. Interviews contextualized the quantitative analysis.

**Results:**

There were 78 928 visits in practices conducting any SDOH screening. The population with visits had a mean (SD) age of 57.6 (18.1) years; 48 086 (60.9%) were female, 12 569 (15.9%) Black, 60 578 (76.8%) White, and 3088 (3.9%) Hispanic. A total of 54 611 visits (69.2%) were with a doctor of medicine and 13 035 (16.5%) with a nurse practitioner. Most had no SDOH questions answered (75 298 [95.4%]) followed by all questions (2976 [3.77%]). Logistic regression analysis found that clinician type, patient race, and primary payer were associated with screening likelihood: for clinician type, nurse practitioner (odds ratio [OR], 0.13; 95% CI, 0.03-0.62; *P* = .01) and physician assistant (OR, 3.11; 95% CI, 1.19-8.10; *P* = .02); for patient race, Asian (OR, 1.69; 95% CI, 1.25-2.28; *P* = .001); Black (OR, 1.49; 95% CI, 1.10-2.01; *P* = .009); or 2 or more races (OR, 1.48; 95% CI, 1.12-1.94; *P* = .006); and for primary payer, Medicaid (OR, 0.62; 95% CI, 0.48-0.80; *P* < .001); managed care (OR, 1.17; 95% CI, 1.07-1.29; *P* = .001); uninsured or with Access Health (OR, 0.26; 95% CI, 0.10-0.67; *P* = .005), and Tricare (OR, 0.71; 95% CI, 0.55-0.92; *P* = .01). Interview themes included barriers (patient hesitancy, time and resources for screening and referrals, and number of questions/content overlap) and facilitators (communication, practice champions, and support for patient needs).

**Conclusions and Relevance:**

This qualitative study presents potential guidance regarding factors that could improve SDOH screening within busy clinical workflows.

## Key Points

**Question:**

Are patient and clinician factors associated with early implementation of social determinants of health (SDOH) screening in primary care, and what strategies can improve these efforts?

**Findings:**

In this qualitative study of 78 928 primary care visits from the inception of primary care–based SDOH screening, visits with a physician assistant, belonging to a racial minority group, and having noncommercial/nonprivate health insurance were associated with greater screening likelihood. Stakeholders suggest that patient-clinician rapport, practice champions, streamlined questions, and referral follow-up ability may improve screening implementation.

**Meaning:**

Results of this study suggest that primary care SDOH screening is feasible but limited by barriers that can be overcome with consideration of stakeholder feedback.

## Teaser

This qualitative analysis uses patient and care team member feedback to identify barriers and facilitators to social determinants of health screening within primary care to inform future screening.

## Introduction

Health systems in the US recognize the importance of social determinants of health (SDOH) in patient outcomes and care. The SDOH are economic and social conditions affecting health outcomes,^1^ health care use,^2^ and health inequities.^3^ Health systems are increasingly engaging in SDOH screening.^4^ Although such screening can potentially improve health outcomes and reduce health care use,^5,6^ there is limited peer-reviewed evidence incorporating patient and clinician or care team characteristics and perspectives when describing early screening initiatives.

Given the personal nature and limited evidence guiding SDOH screening adoption,^7,8,9^ it is critical to understand stakeholder perspectives. Prior research indicates that health care professionals recognize the importance of addressing patient SDOH needs and strive to adopt patient-centered approaches^10^ but face ethical and time-related challenges.^8,11,12^ Existing work reports greater SDOH screening uptake in primary care vs specialist visits and lower completion among patients requiring interpreters and patients with racial and ethnic minority status.^7^ Studies on patient and caregiver perspectives have documented SDOH screening acceptability and preferences.^13^ The role of practice and care team characteristics in screening uptake has not been assessed within a multistakeholder analysis.

To address this research gap, we conducted a qualitative study of a large southeastern US health care system's experiences during the early stages of SDOH screening in primary care. Quantitative analysis examined practice, care team, and patient characteristics and SDOH screening uptake. Qualitative analysis engaged team member feedback. Patient experts informed interview protocols and finding interpretation. Our goal was to identify barriers and facilitators to SDOH screening within primary care to inform future screening.

## Methods

### Study Setting and Population for Quantitative Analysis

This qualitative study was classified as exempt by the Prisma Health institutional review board in accordance with 45 CFR §46. In February 2022, Prisma Health, South Carolina’s largest nonprofit health system with approximately 1.5 million unique patients annually, began screening adults for SDOH needs in primary care practices with the goal of annual screening. Practices had implementation flexibility and determined how and when to screen during the clinical workflow. Patients were screened using a 16-question electronic health record (EHR)–embedded survey (eTable 1 in Supplement 1). Questions were chosen using validated questionnaires and clinical input on system priorities and resource availability. Answers triggered automated input of community-based service information curated to patient SDOH needs and location into patient after-visit summaries using an EHR-compatible platform connecting patients to community-based organizations (NowPow; Unite Us). Practices provided the after-visit summaries to patients at visit end. Reporting follows the 21-item Standards for Reporting Qualitative Research (SRQR) reporting guideline.

The study population included patients aged 18 years or older with a visit in a family or internal medicine practice in the northwestern region of South Carolina from February 22 to May 10, 2022. Visits classified as future, cancelled, no show, or left without being seen were excluded. The last screen on a day was the patient final value, and the same patient could have multiple visits over the study period. In 2021, the northwestern region (4 counties) had 813 069 inhabitants, with 14.2% in poverty (11.4% nationally) and 13.9% uninsured (10.2% nationally). The population is 75.8% White, 14.6% Black, 6.5% Hispanic, 0.4% American Indian or Alaska Native, 1.6% Asian, and 0.1% Native Hawaiian or Other Pacific Islander.^14^

### Analysis

#### Methods for Quantitative Analysis

The primary outcome was SDOH screening completion status. Visits with a response to at least 1 question were deemed partial screening while complete screening included responses to all questions. Our primary outcome compared visits with complete or partial screening (any screening) with no screening. Secondary outcomes compared visits with complete vs partial or no screening and visits with complete screening vs partial screening.

Potential explanatory variables included practice type (family or internal medicine), clinician qualification (medical doctor, doctor of osteopathic medicine, nurse practitioner, and physician assistant), patient demographic characteristics (age, sex, race and ethnicity [treated as classified in the electronic medical records as separate fields], preferred language, primary payer), and SDOH risk (calculated as the ratio of screener questions with positive responses to the total number of questions answered by patients). Race and ethnicity came from the EHR and thus were primarily patient self-reported. Race is reported as Asian, Black, White, 2 or more races, other race, patient refused, or unknown. Other race comprises American Indian or Alaska Native, Native Hawaiian or Other Pacific Islander, and other as reported in the EHR. Ethnicity is reported as in the EHR. We included SDOH risk to test whether patients with a need might be more likely to be screened (ie, care team members suspect a need or patients are more likely to answer questions).

Binary logistic regression was used to determine the odds of screening completion. Standard errors were clustered by practice to account for practice-specific differences. A 95% CI not including 1 indicated statistical significance. We tested for multicollinearity using variance inflation factors and omitted variable bias using the Ramsey Regression Equation Specification Error Test (RESET). Analysis was conducted using Stata/MP, version 11 (StataCorp LLC).

#### Study Setting and Sample for Qualitative Analysis

Six practices were categorized as higher-adopting facilities as they performed SDOH screening during at least 4.0% of visits over the study period. Two of these practices were excluded because of involvement in other SDOH-related studies. Lower-adopting practices performed at least 10 screenings but in less than 2.0% of visits. Four practices met this criterion, but 1 practice was excluded because of involvement in SDOH pilot efforts. Higher- and lower-adopting was defined by quantitative analysis. We excluded practices performing no or minimal screening because we wanted to learn from those practices with some screening familiarity and those screening at both higher and lower levels. These 7 practices were approached for interviews of primary care team members (ie, physicians, administrative staff, nursing staff, and allied health professionals). Six practices participated in a total of 9 interviews (at least 1 interviewee from each of these 6 practices). Interview findings contextualized the quantitative analysis.

#### Methods for Qualitative Analysis

Two trained medical students (E.K. and M.J.) conducted and recorded 9 semistructured interviews online between July 6, 2022, and March 8, 2023. The students had not met the interviewees or worked in these clinics prior to the interviews. Interview questions focused on potential barriers and facilitators to screening (eMethods 1 in Supplement 1). Oral consent was obtained prior to interviews. Interviews were transcribed verbatim by a speech-to-text service (rev.com). Interview recordings were accessible only to interviewers and the team member uploading for transcription. Interviewers asked questions aimed to not yield identifying information. Additionally, transcripts were kept either on secure file-sharing systems or on password-protected computers. Using a web application (Dedoose), transcripts were coded by 2 research team members (D.G. and M.M.) and analyzed using an inductive grounded theory approach, in which important concepts and themes are derived from close reading of the text, and similar concepts are grouped into conceptual categories (codes). No further interviews were necessary as theme saturation was achieved.

#### Patient Engagement

To ensure the research was relevant and ethical for patients and the broader community, we included a meeting with patient experts from the University of South Carolina Patient Engagement Studio (PES) in our research strategy.^15,16,17^ The PES is built on guidance from the Patient-Centered Outcomes Research Institute and provides structured opportunities for research teams to engage with community-recruited patient experts. *Patient expert* refers to individuals or caregivers with substantial health system interaction due to their health conditions who are trained in communication, research methods, and team building.

The research team met with patient experts on June 30, 2022, prior to interviews with primary care practices. In accordance with standard PES processes,^18^ patient experts were provided the health system SDOH screening tool as presession reading material. Discussion topics at that meeting included screening and referral processes (eMethods 2 in Supplement 1). Patient expert feedback was incorporated into the research process through practice interview topics and by incorporating what we heard from patient experts when discussing study results.

## Results

### Descriptive Statistics for Practice Visits

Over the study period, there were 147 096 practice visits, with 3630 (2.5%) involving complete (2976 [3.8%]) or partial (654 [0.8%]) SDOH screening. In the restricted sample, 22 of 58 practices (37.9%) performed any screening during the study period (Table 1). Of the 78 928 visits (mean [SD] age of 57.6 [18.1] years; 48 086 [60.9%] were female, 12 569 [15.9%] Black, 60 578 [76.8%] White and 3088 [3.9%] Hispanic) in the restricted sample, 41 574 (52.7%) were in family medicine and 37 354 (47.3%) in internal medicine practices. Most visits were with medical doctors (54 611[69.2%]), followed by nurse practitioners (13 035 [16.5%]), doctors of osteopathic medicine (5877 [7.4%]), and physician assistants (2958 [3.8%]). On average, patients had a mean (SD) of 0.08 (0.13) (95% CI, 0.08-0.09) positive responses per SDOH question answered.

**Table 1.** Descriptive Statistics for Practice Visits, Restricted Sample^a^ (N = 78 928)

Variable	No. of visits	% (95% CI) [SD]
Practice or clinician demographic characteristics		
Practice specialty		
Family medicine	41 574	52.7 (52.3-53.0) [0.499]
Internal medicine	37 354	47.3 (47.0-47.7) [0.499]
Clinician type		
Doctor of medicine	54 611	69.2 (68.9-69.5) [0.462]
Nurse practitioner	13 035	16.5 (16.3-16.8) [0.371]
Doctor of osteopathic medicine	5877	7.45 (7.26-7.63) [0.263]
Physician assistant	2958	3.75 (3.62-3.88) [0.190]
Unspecified	2302	2.92 (2.80-3.04) [0.168]
Other^b^	145	0.18 (0.16-0.22) [0.043]
Patient demographic characteristics		
Screening completion status		
No screening	75 298	95.4 (95.3-95.5) [0.209]
Complete screening	2976	3.77 (3.64-3.91) [0.190]
Partial screening	654	0.83 (0.77-0.89) [0.091]
SDOH risk, mean (95% CI)^c^	3630	0.08 (0.08-0.09) [0.126^d^]
Age, mean (95% CI), y	78 928	57.6 (57.5-57.8) [18.1^e^]
Sex		
Female	48 086	60.9 (60.6-61.3) [0.488]
Male	30 839	39.1 (38.7-39.4) [0.488]
Unknown/unspecified	3	0.004 (0.001-0.01) [0.006]
Race		
Asian	1070	1.36 (1.28-1.44) [0.116]
Black	12 569	15.9 (15.7-16.2) [0.366]
Patient refused	146	0.19 (0.16-0.22) [0.043]
Unknown	802	1.02 (0.95-1.09) [0.100]
White	60 578	76.8 (76.5-77.0) [0.422]
Other race^f^	3434	4.35 (4.21-4.50) [0.204]
Ethnicity		
Hispanic or Latino	3088	3.91 (3.78-4.05) [0.194]
Non-Hispanic or non-Latino	74 524	94.4 (94.3-94.6) [0.230]
Refused/declined	1314	1.66 (1.58-1.76) [0.128]
Unspecified	2	0.003 (0.000-0.009) [0.005]
Preferred language		
English	77 541	98.2 (98.1-98.3) [0.131]
Spanish	944	1.20 (1.12-1.27) [0.109]
Other	443	0.56 (0.51-0.62) [0.075]
Payer financial class		
Private or commercial	23 616	29.9 (29.6-30.2) [0.458]
Medicare	18 749	23.8 (23.5-24.1) [0.426]
Medicare Advantage	15 653	19.8 (19.6-20.1) [0.399]
Managed care	11 380	14.4 (14.2-14.7) [0.351]
Medicaid	5069	6.42 (6.25-6.60) [0.245]
Missing	3232	4.09 (3.96-4.24) [0.198]
Tricare	653	0.83 (0.77-0.89) [0.091]
Uninsured or AccessHealth	436	0.55 (0.50-0.61) [0.074]
Other^g^	140	0.18 (0.15-0.21) [0.042]

Abbreviation: SDOH, social determinants of health.

^a^ Restricted sample includes visits in only those practices that did any SDOH screening (complete or partial) during the study period.

^b^ Other included certified medical assistants, certified medical laboratory technicians, licensed practical nurse, doctor of philosophy, doctor of pharmacy, registered dietician, licensed dietician, certified diabetes educator, registered medical assistant, registered nurse, and respiratory therapist.

^c^ SDOH risk is calculated as the ratio of SDOH screener questions with positive responses to the total number of questions answered by patients. Positive responses are indicated in eTable 1 in Supplement 1.

^d^ Range, 0-1.

^e^ Range, 18-112.

^f^ Other race includes American Indian or Alaska Native, Native Hawaiian or Other Pacific Islander, and other as reported in the electronic health record.

^g^ Other includes self-pay, liability, pending Medicaid and Worker’s Compensation.

The SDOH screener responses in order of question appearance are given in eTable 1 in Supplement 1. Earlier questions were more likely to be asked and answered. Overall, patient response refusal was low (≤3.3%). Descriptive statistics for the unrestricted sample (visits to all practices) are given in eTable 2 in Supplement 1.

### Factors Associated With SDOH Screening Completion

Table 2 displays regression results examining factors associated with any SDOH screening (complete or partial screening vs no screening) in the restricted (model 1) and unrestricted (model 2) practice samples. In model 1 (restricted), compared with visits with a medical doctor, visits with a physician assistant had 3.11 (95% CI, 1.19-8.10; *P* = .02) greater odds of any screening done, while visits with nurse practitioners had significantly lower odds (odds ratio [OR], 0.13; 95% 0.03-0.62; *P* = .01) of any screening done. Visits with patients identifying as Asian (OR, 1.69; 95% CI, 1.25-2.28; *P* = .001), Black (OR, 1.49; 95% CI, 1.10-2.01; *P* = .009), or 2 or more races (OR, 1.48; 95% CI, 1.12-1.94; *P* = .006) were more likely to have any screening compared with visits with patients identifying as White. With regard to primary payer, visits where patients had managed care had 1.17 (95% CI, 1.07-1.29; *P* = .001) greater odds of any screening compared to visits where patients had private or commercial payers. Visits where patients had Medicaid (OR, 0.62; 95% CI, 0.48-0.80; *P* < .001), were uninsured or had Access Health (OR, 0.26; 95% CI, 0.10-0.67; *P* = .005) or had Tricare (OR, 0.71; 95% CI, 0.55-0.92; *P* = .01) had lower odds of any screening. Practice type, patient age, sex, language, and ethnicity had no significant associations with screening likelihood. Results were consistent in model 2 (unrestricted) except for visits with physician assistants and uninsured patients, where the finding was not significant.

**Table 2.** Multivariable Logistic Regression Examining Associations With Any SDOH Screening (Complete or Partial) vs No SDOH Screening

Variable	Any screening (complete/partial) vs no screening			
	Model 1 (restricted model)^a^^,^^b^		Model 2 (unrestricted model)^c^	
	Odds ratio (95% CI)	*P* value	Odds ratio (95% CI)	*P* value
Practice type				
Family medicine	1 [Reference]	NA	1 [Reference]	NA
Internal medicine	0.43 (0.10-1.94)	.27	0.57 (0.11-2.86)	.50
Clinician type				
Doctor of medicine	1 [Reference]	NA	1 [Reference]	NA
Doctor of osteopathic medicine	1.66 (0.83-3.32)	.15	0.98 (0.57-1.68)	.95
Nurse practitioner	0.13 (0.03-0.62)	.01	0.10 (0.02-0.49)^e^	.004
Physician assistant	3.11 (1.19-8.10)	.02	1.79 (0.75-4.30)	.19
Age (centered)	1.00 (0.99-1.00)	.32	1.00 (0.99-1.01)	.68
Sex				
Female	0.93 (0.85-1.01)	.09	0.92 (0.85-1.00)	.05
Male	1 [Reference]	NA	1 [Reference]	NA
Preferred language				
English	1 [Reference]	NA	1 [Reference]	NA
Spanish	1.08 (0.53-2.18)	.83	1.15 (0.57-2.30)	.70
Other	1.05 (0.67-1.64)	.83	1.15 (0.79-1.68)	.46
Race				
Asian	1.69 (1.25-2.28)	.001	1.98 (1.37-2.87)	<.001
Black	1.49 (1.10-2.01)	.009	1.95 (1.39-2.74)	<.001
≥2 Races	1.48 (1.12-1.94)	.006	1.61 (1.18-2.20)	.003
White	1 [Reference]	NA	1 [Reference]	NA
Other race^d^	1.23 (0.86-1.77)	.26	1.36 (0.95-1.97)	.10
Ethnicity				
Hispanic or Latino	1.09 (0.91-1.32)	.34	1.21 (0.98-1.51)	.08
Non-Hispanic or non-Latino	1 [Reference]	NA	1 [Reference]	NA
Payer financial class				
Private or commercial	1 [Reference]	NA	1 [Reference]	NA
Medicaid	0.62 (0.48-0.80)	<.001	0.67 (0.53-0.85)	.001
Medicare	1.19 (0.67-2.14)	.51	1.17 (0.66-2.07)	.59
Medicare Advantage	1.11 (0.56-2.22)	.76	1.06 (0.53-2.10)	.87
Managed care	1.17 (1.07-1.29)	.001	1.17 (1.06-1.29)	.002
Uninsured or Access Health	0.26 (0.10-0.67)	.005	0.35 (0.11-1.09)	.07
Tricare	0.71 (0.55-0.92)	.01	0.65 (0.49-0.87)	.003
Constant	0.07 (0.01-0.31)	.001	0.03 (0.007-0.15)	<.001

Abbreviations: NA, not applicable; SDOH, social determinants of health.

^a^ Restricted model includes only those observations from practices that had at least 1 SDOH screening occurrence (partial or full).

^b^ For model 1, there were 76 621 observations. The pseudo *R*^2^ value was 0.071; the Regression Equation Specification Error Test (RESET) value was 3.60; and the probability >χ2 value was 0.166.

^c^ For model 2, there were 144 199 observations. The Wald χ^2^_27_ value was 583.4; the probability >χ2 value was <.001; the pseudo *R*^2^ value was 0.056; the RESET value was 4.33; and the probability >χ2 value was 0.115.

^d^ Other race includes American Indian or Alaska Native, Native Hawaiian or Other Pacific Islander, and other as reported in the electronic health record.

We also compared visits completing the entire screening questionnaire vs partial or no screening (Table 3) for the restricted practice sample. In model 3, compared with visits with a medical doctor, visits with a physician assistant had 3.78 times (95% CI; 1.43-10.0; *P* = .007) greater odds of screening completion while visits with a nurse practitioner had lower screening completion odds (OR, 0.15; 95% CI, 0.03-0.75; *P* = .02). Visits where patients identified as Black had greater odds of screening completion (OR, 1.33; 95% CI, 1.01-1.74; *P* = .04) than visits where patients identified as White. Visits where patients had managed care had 1.15 (95% CI, 1.05-1.26; *P* = .002) times greater screening completion odds than visits where patients had private or commercial payers. However, screenings were less likely to be complete if patients had Medicaid (OR, 0.53; 95% CI, 0.40-0.72; *P* < .001), Tricare (OR, 0.76; 95% CI, 0.58-0.98; *P* = .04), or were uninsured or had Access Health (OR, 0.14; 95% CI, 0.05-0.40; *P* < .001). Results were consistent in model 4 comparing the odds of complete vs partial screening.

**Table 3.** Multivariable Logistic Regression Examining Associations With Complete SDOH Screening vs Partial or No SDOH Screening

Variable	Model 3, complete screening vs partial or no screening (restricted model)^a^		Complete screening vs partial screening			
			Model 4 (restricted model)^b^		Model 5 (restricted model, includes SDOH risk)^c^	
	Odds ratio (95% CI)	*P* value	Odds ratio (95% CI)	*P* value	Odds ratio (95% CI)	*P* value
Practice type						
Family medicine	1 [Reference]	NA	1 [Reference]	NA	1 [Reference]	NA
Internal medicine	0.31 (0.06-1.74)	.18	0.26 (0.03-2.00)	.19	0.26 (0.03-1.95)	.19
Clinician type						
Doctor of medicine	1 [Reference]	NA	1 [Reference]	NA	1 [Reference]	NA
Doctor of osteopathic medicine	1.88 (0.93-3.80)	.08	1.39 (1.09-1.78)	.008	1.39 (1.08-1.79)	.01
Nurse practitioner	0.15 (0.03-0.75)	.02	1.59 (0.76-3.31)	.22	1.59 (0.77-3.29)	.22
Physician assistant	3.78 (1.43-10.0)	.007	4.17 (1.79-9.75)	.001	4.17 (1.78-9.77)	.001
Age (centered)	1.00 (0.99-1.00)	.61	1.02 (1.00-1.03)	.09	1.02 (1.00-1.04)	.10
Sex						
Female	0.92 (0.84-1.01)	.07	0.88 (0.78-0.99)	.03	0.88 (0.78-1.00)	.05
Male	1 [Reference]	NA	1 [Reference]	NA	1 [Reference]	NA
Preferred language						
English	1 [Reference]	NA	1 [Reference]	NA	1 [Reference]	NA
Spanish	1.21 (0.45-3.23)	.71	1.83 (0.14-23.6)	.64	1.83 (0.14-23.3)	.64
Other	0.92 (0.62-1.37)	.69	0.58 (0.36-0.93)	.03	0.58 (0.36-0.93)	.03
Race						
Asian	1.45 (0.92-2.29)	.11	0.45 (0.20-1.04)	.06	0.45 (0.20-1.04)	.06
Black	1.33 (1.01-1.74)	.04	0.56 (0.27-1.16)	.12	0.56 (0.28-1.14)	.11
≥2 Races	1.11 (0.89-1.39)	.35	0.25 (0.12-0.52)	<.001	0.25 (0.12-0.51)	<.001
White	1 [Reference]	NA	1 [Reference]	NA	1 [Reference]	NA
Unknown	1.34 (1.00-1.80)	.05	0.58 (0.27-1.22)	.15	0.58 (0.27-1.23)	.16
Other race^d^	1.01 (0.79-1.28)	.96	0.47 (0.30-0.73)	.001	0.47 (0.30-0.72)	.001
Ethnicity						
Hispanic or Latino	1.19 (0.98-1.45)	.09	1.20 (0.83-1.73)	.34	1.20 (0.82-1.74)	.35
Non-Hispanic or non-Latino	1 [Reference]	NA	1 [Reference]	NA	1 [Reference]	NA
Payer financial class						
Private or commercial	1 [Reference]	NA	1 [Reference]	NA	1 [Reference]	NA
Medicaid	0.53 (0.40-0.72)	<.001	0.36 (0.21-0.62)	<.001	0.36 (0.21-0.61)	<.001
Medicare	0.95 (0.68-1.34)	.76	0.28 (0.11-0.69)	.006	0.28 (0.12-0.69)	.005
Medicare Advantage	0.88 (0.53-1.47)	.63	0.28 (0.10-0.75)	.01	0.28 (0.10-0.73)	.01
Managed Care	1.15 (1.05-1.26)	.002	0.84 (0.73-0.97)	.02	0.84 (0.73-0.97)	.02
Uninsured or Access Health	0.14 (0.05-0.40)	<.001	0.08 (0.03-0.23)	<.001	0.08 (0.03-0.23)	<.001
Tricare	0.76 (0.58-0.98)	.04	2.63 (0.46-15.2)	.28	2.63 (0.46-15.2)	.28
SDOH risk^e^	NA	NA	NA	NA	1.03 (0.56-1.88)	.93
Constant	0.06 (0.01-0.29)	<.001	18.5 (7.68-44.6)	<.001	18.5 (7.58-45.1)	<.001

Abbreviations: NA, not applicable; SDOH, social determinants of health.

^a^ For model 3, there were 76 621 observations. The pseudo *R*^2^ value was 0.089; the Regression Equation Specification Error Test (RESET) value was 4.06; and the probability >χ2 value was 0.132.

^b^ For model 4, there were 3629 observations. The pseudo *R*^2^ value was 0.140; the RESET value was 16.64; and the probability >χ2 value was <.0012.

^c^ For model 5, there were 3629 observations. The pseudo *R*^2^ value was 0.140; the RESET value was 15.64; and the probability >χ2 value was 0.000.

^d^ Other race includes American Indian or Alaska Native, Native Hawaiian or Other Pacific Islander, and other as reported in the electronic health record.

^e^ SDOH risk is calculated as the ratio of SDOH screener questions with positive responses to the total number of questions answered by patients. Positive responses are indicated in eTable 1 in Supplement 1.

Model 5 extended model 4 to include patient SDOH risk from screening responses. Patient SDOH risk was not associated with screening completion (OR, 1.03; 95% CI, 0.56-1.88; *P* = .93). Results in model 5 are consistent with model 4.

All models had variance inflation factors of less than 10 indicating absence of multicollinearity. Models 4 and 5 had omitted variable bias.

### Health Care Team Member Experience

#### Barriers

We identified 7 themes regarding barriers and facilitators from health care team member interviews for implementing SDOH screening (Table 4). Care team members reported patient reluctance in responding to screener questions. Hesitancy was attributed to perceptions about questions being intrusive or offensive. Interviewees reported patients reacting unfavorably to sensitive questions (eg, violence/abuse, financial strain). Time to administer the screener, interpret results, and address identified needs posed challenges with existing workloads.

**Table 4.** Themes and Quotations From Primary Care Practice Interviews

Theme	Example quotation
**Barriers to implementing SDOH screening**	
Patient perceptions about SDOH screening	“Let’s see, we’ve never really asked about the finance stuff, since I’ve worked here, so that’s one of the things that I find patients really do not like. They find that very offensive. Then, housing stuff, I don’t think that’s really included, but the abuse stuff we ask, the safety section, let’s see, the social stuff about friends and family. Some people find that weird to ask, is what I’ve been told.”
Clinician time constraints for screening	“As a provider, I’ve been trying sometimes to pick up the paper as time allows to kind of go through the questions or even put it in myself as opposed to my nurses, traditionally the nurses put it in, so I can catch anything to talk about. But it’s really hard. We have lots and lots and lots of demands on us and this is just another thing, so we have multiple things. And so I would say that’s the hardest part about this, is it’s just another thing. We don’t have a visit for social determinants of health. We have visits for about 20 things we’re trying to accomplish.”
Number of questions and content overlap	“The last 4 questions…because, I mean, ‘Are you afraid of your partner?’, ‘Have you been humiliated or emotionally abused?’, ‘Have you been kicked, hit, slapped, physically hurt?’. Do you see what I’m saying? So, all those safety questions. And then it goes into the raped or forced to have... I mean, I think those questions in itself, maybe there would be a different way. That’s just my question. It just seems like it just goes on and on and on, you know? Question after question, is what I’m saying.”
Training and resources for implementing SDOH screening and referrals	“I would have to say it could be better if our clinical teams and our providers all had some kind of, I don’t know, in-service training around things like this. When you implement them, that they would exactly know the why behind it and exactly what you do. In other words, for me, it’s like, ‘Why? Where did this come from? What is it that we need to do, and what happens once we do it? And where do we get all of these resources?’ So I do think if you had all of that answered upfront, people could do far better with all of it.”
**Facilitators to encourage SDOH screening**	
Focusing on patient-clinician communication	“The biggest thing that’s worked well for us was having almost 2 ways and we can kind of ask consent from the patient, ‘Would you feel comfortable verbally answering these questions?’ Because some people, their reading comprehension varies a little bit but also helps because I said there’s been some misunderstanding with some of the questions too at times. But giving them the opportunity even if they fill it out by a form that they can ask questions about if they need to.”
Having practice champions	“So, nurse in our office can also mean CMAs, certified medical assistants. So, the RNs and CMAs are the main drivers, and then the providers theoretically review it and look at it and make sure it’s put in afterwards. But usually, it really is our nurses that are doing it ultimately. They’re the ones that are doing it. And I say that because sometimes in our business, the providers may not even see the answer to the questions. So that’s a part of the system that’s probably not great, but it’s just the reality of a busy outpatient practice.”
Enhancing support for patient SDOH needs	“Our providers certainly don’t need training on referrals and how to refer patients out for assistance. I think my question is, when you say, ‘Did you follow up on that?’ That’s where I think we would have to have some additional training. And additional help. And additional staffing in order to accommodate closing that loop.”

Abbreviations: RN, registered nurse; SDOH, social determinants of health.

Clinicians expressed concerns about potential patient response burden and overlap with routine care questions (eg, stress and Patient Health Questionnaire 2). Clinicians suggested streamlining the screener by combining multiple related questions and then tailoring subsequent questions based on patient initial responses.

Some clinicians felt inadequately trained in navigating the screening tool and expressed uncertainty about effective use of screening results. Many practices lacked social workers or resource navigators to connect patients with resources and follow up on referrals. Clinicians felt their attention diverted from the primary goal of medical care provision.

#### Facilitators

Care team members reported that screening facilitated patient care by uncovering socioeconomic issues not identified in routine care. Practices that informed patients about the screening purpose, assured them it would not affect care, and obtained verbal consent prior to screener administration perceived more successful uptake.

Some practices identified practice champions as being responsible for screening implementation and supporting patient needs. Some practices had a referral coordinator or social worker who connected patients to community-based resources and provided follow-up support. Clinicians reported they would benefit from training on how to best use screening.

### Patient Expert Feedback on SDOH Screening Implementation

Table 5 presents feedback from patient experts. Patient experts preferred that screening be done at annual appointments to allow for discussion time and in the examination room to ensure privacy. Patient experts emphasized rapport building between patients and care teams and providing information about the screening purpose. They expressed the importance of empathetic clinicians performing screening. Recommendations for rephrasing questions included expanding the partner violence or abuse questions (eTable 2 in Supplement 1) to include safety concerns related to family members, neighborhoods, and caretakers. Patient experts expressed concern about timely referral follow-up.

**Table 5.** Quotations From Engagement With Patient and Community Stakeholders

Theme	Example quotation
SDOH screening appointments	“I can see this fitting in best, like, in an annual physical appointment that’s a little longer.”
SDOH screening location	“Environment for the most honest feedback will be actually inside the doctor’s office.”
Patient-clinician rapport building	”Because if you sat me down in a room with someone I’ve never seen before and they start giving this questionnaire...you’re not going to get truthful answers out of people.”
Phrasing of SDOH questions	“You should elaborate a little bit more because there’s so many ways of being abused other than physical.”
Following up after referrals	“Somebody will contact you. I like it because then it’s not up to the person to drive the process. The system will make sure that somebody’s addressing the person.”

Abbreviation: SDOH, social determinants of health.

## Discussion

This qualitative study assessed factors associated with SDOH screening completion in primary care and explored patient and care team member perspectives on screening. We found that clinician type, patient race, and primary payer were linked to any screening but that practice type, patient age, sex, language, ethnicity and SDOH risk were not.

Completion rates differed in this study (3.8%) from previous research (58.7%)^7^ also examining systemwide SDOH screening implementation. This may be related to study duration, timing (intra–COVID-19 pandemic vs pre–COVID-19 pandemic), or implementation (recommendation for all primary care patients vs preassigned screening).^7^ Based on qualitative interviews, our study completion rates may be affected by the desire to receive more resources to support patient referrals.

Our findings suggest that primary care visits with nonphysician clinicians, such as physician assistants, may be favorable for SDOH screening. However, this result did not hold for nurse practitioners and deserves further research, as previous studies demonstrated nonphysician clinician confidence in addressing SDOH needs and greater community-based resource awareness.^19^ Clinician type could be serving as a proxy for visit type as our data set did not include visit reason. Consistent with previous studies,^20^ our interview-based findings suggest that clinicians faced an additional time burden from incorporating SDOH screening, which they perceived to affect care provision.

We found patients with managed care to be more likely to be screened, while those with Medicaid and those who were uninsured or had Access Health and Tricare were less likely. Medicare and Medicare Advantage had no effect relative to private or commercial payer status. Patients with Medicaid and uninsured or had Access Health may benefit most from screening; therefore this finding is critical for further implementation. Of note, these patients may have been screened via other programs at the health system thus, lack of screening in primary care is not necessarily reflective of screening otherwise.

A lack of association between screening and other patient characteristics (age, gender, language, ethnicity, SDOH risk) suggests that perhaps these characteristics are not associated with SDOH needs in the perceptions of those performing screening. These results differed from previous research that found members of racial and ethnic minority groups less likely to be screened,^7^ thereby providing support for universal implementation across primary care practices as a potential mitigation against screening disparities.^7^

In our quantitative analysis, questions appearing later in the screener were less likely to be completed. Interviews further explained this finding as questionnaire length and repetitive questions led to a greater perceived patient response burden by health care clinicians. Although there is no consensus on screener length, existing tools range from 6 to 23 questions.^21^ Generally, short-form surveys are more acceptable to patients.^22^ Notably, patients did not express the same concerns as clinicians about survey length or repetitiveness.

Interviews and patient expert feedback found that patient–care team communication is crucial for screener uptake. Sensitive questions about patient needs may lead to incomplete or untruthful responses if patients have privacy concerns,^10,23^ feel embarrassed, or fear stigmatization.^24^ Patient experts and health care team members emphasized rapport building and communicating the screening purpose to mitigate patient concerns and build trust. Future investigation should include assessment of standard phrasing to introduce the screener rationale and consideration of the best location and visit type for screening. Last, patient experts and care team members expressed concerns about referral follow-up, perceiving that care would benefit from an enhanced ability to follow up on referral outcomes.

### Limitations

Our study has a few limitations to be considered. First, findings are restricted to primary care practices within 1 health system in 1 region, limiting generalizability. However, this study is comprehensive by including all primary care practices in 1 region covered by a large health system that statewide serves approximately 25% of residents.^14^ Second, we used a convenience sample of practice staff for our qualitative assessment. This restricted our examination of how qualitative themes differed based on practice characteristics. However, practice choice for interviews was based on screening implementation to intentionally capture those screening at higher and lower adoption rates. Third, our data set included whether a survey was taken on MyChart (Epic). No surveys were done on MyChart. Accordingly, we were unable to test screening modality association with screening completion. We also had no information on screening completion via telemedicine vs office visits and did not include this topic in our interview guide. In addition, we do not know at what rate patients refused to verbally consent to screener administration if a practice asked for such consent.

## Conclusions

Although health systems face different challenges in implementing SDOH screening, identifying and addressing common barriers are critical for improved patient activation and care collaboration. Future research should focus on robust assessment of strategies to improve screening uptake.

## Notes

Supplement 1. **eMethods 1.** Semi-Structured Interview Guide for Primary Care Team Members **eMethods 2.** Discussion Questions for Patient Engagement Studio (PES) With Patient Stakeholders **eTable 1.** Descriptive Statistics for Social Determinants of Health (SDOH) Screening Responses **eTable 2.** Descriptive Statistics for Practices, Providers and Patients With Unrestricted Sample (N = 147 096)

Supplement 2.
Data Sharing Statement
