# Supplementary material for: Patient and Care Team Perspectives on Social Determinants of Health Screening in Primary Care: A Qualitative Study
Source: JAMA Netw Open. 2023 Nov 28;6(11):e2345444. doi: 10.1001/jamanetworkopen.2023.45444 (PMC10685887; doi:10.1001/jamanetworkopen.2023.45444)
Supplement: Supplement 1. — eMethods 1. Semi-Structured Interview Guide for Primary Care Team Members eMethods 2. Discussion Questions for Patient Engagement Studio (PES) With Patient Stakeholders eTable 1. Descriptive Statistics for Social Determinants of Health (SDOH) Screening Responses eTable 2. Descriptive Statistics for Practices, Providers and Patients With Unrestricted Sample (N = 147 096) [file jamanetwopen-e2345444-s001.pdf]

Supplemental Online Content

Rudisill AC, Eicken MGA, Gupta D, et al. Patient and care team perspectives on social determinants of health screening in primary care: a qualitative study. *JAMA Netw Open.* 2023;6(11):e2345444. doi:10.1001/jamanetworkopen.2023.45444

eMethods 1. Semi-Structured Interview Guide for Primary Care Team Members

eMethods 2. Discussion Questions for Patient Engagement Studio (PES) With Patient Stakeholders

eTable 1. Descriptive Statistics for Social Determinants Of Health (SDOH) Screening Responses

eTable 2. Descriptive Statistics for Practices, Providers and Patients With Unrestricted Sample (N=147,096)

eReferences

This supplemental material has been provided by the authors to give readers additional information about their work.

© 2023 Rudisill AC et al. *JAMA Network Open*.

eMethods 1. Semi-structured interview guide for primary care team members

*Interview guide and consent passage***.** *These paragraphs must be read verbatim prior to the start of the interview.*

Thank you for agreeing to participate in this short interview. Prisma Health began screening for social determinants of health in primary care settings over the past few months. We know that each practice has taken its own approach and met different successes and challenges. We want to understand these experiences. We will be asking about how screening for Social Determinants of Health (SDOH) has been implemented in your practice, your views on the utility of screening, and some of the successes and challenges that have occurred.

The information collected during this interview will be used for research purposes. Your responses will be coded, and no one will be able to link responses back to you. Participation in this project is voluntary and a decision not to participate will not impact your employment status, any performance assessments nor will it be reported to your supervisors. You may choose not to answer any questions or end the interview at any time. Your continued participation in this conversation will indicate that you agree to participate in this interview.

Please remember that you do not have to answer any questions that you are not comfortable answering, and you can withdraw your participation at any time without any negative consequences. We will keep your name confidential and any references to this interview in research output will not include your name or any identifying information. We would like to record this interview, and have it transcribed, so that we are sure we don't miss anything important. Is it OK if we do so? If not, then I will take notes. Do you have any questions before we begin? Do you agree to participate in this interview?

1. To start off, we'd like to know a little more about you:
    a. What's your profession?
    b. How many years have you been working in this practice?
    c. What role do you currently have in this practice?
    d. What is your role in implementation of SDOH screening in your center?
2. What is your general opinion of using SDOH information as part of clinical practice?
    a. What is the history, in your clinic, of considering or assessing SDOH with respect to patient health outcomes?
3. How well has the screening for SDOH been integrated into your clinical setting?
    a. What does the current workflow look like–from start to finish–for the SDOH screening process in your clinic?
        i. Who has been the primary team member(s) in charge of executing the SDOH screening process? Is there a practice champion?

© 2023 Rudisill AC et al. *JAMA Network Open*.

ii. When patients are identified as needing resources, how does your practice address this?

iii. Following resource referral, is there someone within the clinic able to follow up with patients?

b. What have been the limiting factors, if any, preventing successful uptake of the tool into clinical workflow?

c. What suggestions do you have relative to successful SDOH screening tool uptake into the clinic?

4. In your experience, do you feel that Prisma Health's SDOH screening tool adequately screens for all areas of potential SDOH disparity?

   a. If not, what areas do you feel require further investigation?

   b. Are there any variables that you feel the SDOH screening tool addresses well, or over-addresses?

   c. What other thoughts do you have relative to the tool specifically?

   d. Are there any instances that stand out where the use of the SDOH screening tool negatively impacted health and/or patient experience or your experience providing care to a patient?

   e. Are there any instances that stand out where the use of the SDOH screening tool positively impacted health and/or patient experience or your experience providing care to a patient?

5. Do you feel like you had enough training prior to SDOH screening getting started in your clinic?

   a. About how to handle patient needs? Where to refer them?

   b. About using the tool within EPIC?

6. Following the integration of the SDOH screening tool into your clinic's workflow, what aspect of the tool has yielded the greatest benefit?

7. Do you have anything else you would like us to know about the use of the SDOH Screening tool, or your thoughts on SDOH more generally?

"Are there any other team members in the practice such as RNs, CNAs, front office staff, clinicians, or anyone else who might be involved or potentially involved in SDOH screening at your practice who we could reach out to?" If they say yes, get the names of anyone they recommend, share these names with xxx*, and xxx* will contact them with our recruitment script.

*Name redacted for author anonymity in peer review

© 2023 Rudisill AC et al. *JAMA Network Open*.

eMethods 2. Discussion questions for patient engagement studio (PES) with patient stakeholders

In the session, the SDOH screening process was described to the patient experts who were then asked the following specific questions related to their perspectives about the process:

(1) What are your suggestions for the best way to have patients complete the screening

(2) Do you think it encompasses all areas of social determinants of health or needs that impact your health outside of traditional healthcare? If not, what is missing?

(3) What do you think is the best way to link patients to resources based on their responses to the screening and what should be done with the data from the patient's responses?

(4) Do you have any other thoughts about the SDOH screening tool and process that you would like for us to know?

© 2023 Rudisill AC et al. *JAMA Network Open*.

eTable 1. Descriptive statistics for social determinants of health (SDOH) screening responses[a]

SDoH screening questions and response options	N	% (95%CI)	Std. Dev.
Food insecurity			
Within the past 12 months, we worried that our food would run out before we got money to buy more[b]	3,581		
Never true	3,181	88.8 (87.8-89.8)	0.315
<u>Often true</u>	59	1.65 (1.26-2.12)	0.127
<u>Sometimes true</u>	242	6.76 (5.96-7.63)	0.251
Patient refused	99	2.76 (2.25-3.36)	0.164
Within the past 12 months, the food we bought just didn't last and we didn't have money to get more[b]	3,581		
Never true	3,238	90.4 (89.4-91.4)	0.294
<u>Often true</u>	51	1.42 (1.06-1.87)	0.119
<u>Sometimes true</u>	192	5.36 (4.65-6.15)	0.225
Patient refused	100	2.79 (2.28-3.39)	0.165
Financial strain			
How hard is it for you to pay for the very basics like food, housing, medical care, and heating?[c]	3,586		
<u>Very hard</u>	42	1.17 (0.845-1.58)	0.108
<u>Hard</u>	82	2.29 (1.82-2.83)	0.149
<u>Somewhat hard</u>	366	10.2 (9.23-11.2)	0.303
Not very hard	726	20.2 (18.9-21.6)	0.402
Not hard at all	2,276	63.5 (61.9-65.0)	0.482
Patient refused	94	2.62 (2.12-3.20)	0.160
Was there a time in the past 12 months when you needed to see a doctor or buy medications but could not because of cost?[d]	3,494		

© 2023 Rudisill AC et al. *JAMA Network Open*.

No	3,120	89.3 (88.2-90.3)	0.309
Yes	283	8.10 (7.22-9.05)	0.273
Patient refused	91	2.60 (2.10-3.19)	0.159
Lack of transportation			
In the past 12 months, has the lack of transportation kept you from medical appointments or from getting medications?[e]	3,576		
No	3,367	94.2 (93.3-94.9)	0.235
Yes	117	3.27 (2.71-3.91)	0.178
Patient refused	92	2.57 (2.08-3.15)	0.158
Stress			
Do you feel tense, restless, nervous, or anxious most days?[f]	3,192		
Very much	126	3.95 (3.30-4.68)	0.195
Quite a bit	233	7.30 (6.42-8.26)	0.260
To some extent	474	14.8 (13.6-16.1)	0.356
Only a little	818	25.6 (24.1-27.2)	0.437
Not at all	1,454	45.6 (43.8-47.3)	0.498
Patient refused	87	2.73 (2.22-3.35)	0.163
*Social isolation**			
In a typical week, how many times do you talk/text with family, friends, or neighbors? [g]	3,206		
More than three times a week	2,368	73.9 (72.3-75.4)	0.439
Three times a week	324	10.1 (9.08-11.2)	0.301
Twice a week	186	5.80 (5.02-6.67)	0.234
Once a week	184	5.74 (4.96-6.60)	0.233
Never	55	1.72 (1.29-2.23)	0.130
Patient refused	89	2.78	0.164

© 2023 Rudisill AC et al. *JAMA Network Open*.

		(2.24-3.41)	
How often do you get together with friends or relatives?[g]	3,198		
More than three times a week	1,001	31.3 (29.7-32.9)	0.464
Three times a week	422	13.2 (12.0-14.4)	0.338
Twice a week	519	16.2 (15.0-17.6)	0.369
Once a week	986	30.8 (29.2-32.5)	0.462
Never	177	5.53 (4.77-6.38)	0.229
Patient refused	93	2.91 (2.35-3.55)	0.168
Violence/abuse			
Within the last year, have you been afraid of your partner or ex-partner?[h]	3,197		
No	3,074	96.1 (95.4-96.8)	0.192
Yes	21	0.66 (0.41-1.00)	0.081
Patient refused	102	3.19 (2.61-3.86)	0.176
Within the last year, have you been humiliated or emotionally abused in other ways by your partner or ex-partner?[h]	3,199		
No	3,058	95.6 (94.8-96.3)	0.205
Yes	34	1.06 (0.74-1.48)	0.103
Patient refused	107	3.34 (2.75-4.03)	0.180
Within the last year, have you been kicked, hit, slapped, or otherwise physically hurt by your partner or ex-partner?[h]	3,189		
No	3,071	96.3 (95.6-96.9)	0.189
Yes	13	0.41 (0.22-0.70)	0.064
Patient refused	105	3.29 (2.70-3.97)	0.178

© 2023 Rudisill AC et al. *JAMA Network Open*.

Within the last year, have you been raped or forced to have any kind of sexual activity by your partner or ex-partner? [h]	3,184		
No	3,074	96.5 (95.9-97.2)	0.183
Yes	4	0.13 (0.03-0.32)	0.035
Patient refused	106	3.33 (2.73-4.01)	0.179
Housing insecurity			
In the last 12 months, was there a time when you did not have a steady place to sleep or slept in a shelter (including now)? [i]	3,170		
No	3,035	95.7 (95.0-96.4)	0.202
Yes	34	1.07 (0.77-1.50)	0.103
Patient refused	101	3.19 (2.63-3.86)	0.176
Are you worried that the place you are living now is making you sick? (has mold, bugs/rodents, water leaks, not enough heat or air conditioning)? [j]	3,155		
No	2,999	95.1 (94.2-95.8)	0.217
Yes	53	1.68 (1.26-2.19)	0.129
Patient refused	103	3.26 (2.67-3.95)	0.178
Exercise level of effort [k]			
How many days per week do you engage in Moderate to Strenuous Exercise? (such as brisk walk)			
On average, how many minutes to you exercise per day			
Total minutes of exercise per week			

[a]Underlined responses to indicate a positive screen for the respective SDoH need

[*] **Note:** Positive screen for social isolation is calculated if the sum of responses to both questions is less than 3, according to the HealthBegins Upstream Risks Screening Tool [6]

SDoH questions obtained/adapted from following sources: [b]Hunger Vital Sign [1]; [c]Accountable Health Communities Health-Related Social Needs Screening Tool [2]; [d]Health Leads Screening Toolkit [3]; [e]Protocol for Responding to and Assessing Patients' Assets, Risks, and Experiences [4]; [f]Accountable Health Communities Health-Related Social Needs Screening Tool [5]; [g]HealthBegins Upstream Risks Screening Tool [6]; [h]Humiliation, Afraid, Rape, Kick (HARK) screening tool [7]; [i]Housing Stability Vital Sign

© 2023 Rudisill AC et al. *JAMA Network Open*.

[8]; [j]Montefiore's 10-Question SDOH Survey [9]; [k]Exercise Vital Sign [10]. These questions are included in the SDOH tool in the EHR but not assessed for the purpose of this study.

© 2023 Rudisill AC et al. *JAMA Network Open*.

eTable 2: Descriptive statistics for practices, providers and patients with unrestricted sample (N=147,096)

Variables	N	% (95% CI)	Std. Dev.	Range[a]
**Practice/provider demographics**				
Practice specialty				
Family medicine	86,739	59.0 (58.7-59.2)	0.492	
Internal medicine	60,357	41.0 (40.8-41.3)	0.492	
Provider title				
Doctor of Medicine (MD)	91,329	62.1 (61.8-62.3)	0.485	
Nurse Practitioner (NP)	29,674	20.2 (20.0-20.4)	0.401	
Doctor of Osteopathic Medicine (DO)	15,273	10.4 (10.2-10.5)	0.305	
Physician Assistant (PA)	7,498	5.10 (4.99-5.21)	0.220	
Unspecified	2,889	1.96 (1.89-2.04)	0.139	
Other[b]	433	0.29 (0.27-0.32)	0.054	
**Patient demographics**				
Screening completion status				
No screening	143,466	97.5 (97.5-97.6)	0.155	
Complete screening	2,976	2.02 (1.95-2.10)	0.141	
Partial screening	654	0.45 (0.41-0.48)	0.067	
SDOH risk[c], mean (95% CI)	3630	0.08 (0.08-0.09)[d]	0.126	0-1
Age, mean (95% CI), years	147,096	56.4 (56.3-56.5)[d]	18.4	18-112
Gender				
Female	90,015	61.2 (60.9-61.4)	0.487	
Male	57,078	38.8 (38.6-39.1)	0.487	

© 2023 Rudisill AC et al. *JAMA Network Open*.

Unknown/Unspecified	3	0.002 (0.000-0.006)	0.005
Race			
Asian	1,702	1.16 (1.10-1.21)	0.107
Black/African American	20,581	14.0 (13.8-14.2)	0.347
Patient refused	282	0.19 (0.17-0.22)	0.044
Two or more races	643	0.44 (0.40-0.47)	0.066
Unknown	1,515	1.03 (0.98-1.08)	0.101
White	116,464	79.2 (79.0-79.4)	0.406
Other race	5,909	4.02 (3.92-4.12)	0.196
Ethnicity			
Hispanic or Latino	5,308	3.61 (3.51-3.71)	0.187
Non-Hispanic or Non-Latino	139,490	94.8 (94.7-94.9)	0.221
Refused/Declined	2,293	1.56 (1.50-1.62)	0.124
Unspecified	5	0.003 (0.001-0.008)	0.006
Preferred language			
English	144,772	98.4 (98.4-98.5)	0.125
Spanish	1,570	1.07 (1.02-1.12)	0.103
Other	754	0.51 (0.48-0.55)	0.071
Payor financial class			
Private/Commercial	45,460	30.9 (30.7-31.1)	0.462
Medicare	32,261	21.9 (21.7-22.1)	0.414
Medicare Advantage	29,236	19.9 (19.7-20.1)	0.399
Managed Care	22,216	15.1 (14.9-15.3)	0.358

© 2023 Rudisill AC et al. *JAMA Network Open*.

Medicaid	10,138	6.89 (6.76-7.02)	0.253
Missing	5,623	3.82 (3.73-3.92)	0.192
Tricare	1,367	0.93 (0.88-0.98)	0.096
Uninsured/Access Health	611	0.42 (0.38-0.45)	0.064
Others[e]	184	0.13 (0.11-0.15)	0.035

[a]Range is provided for continuous variables only
[b]'Other' category includes Certified Medical Assistants (CMA), Certified Medical Laboratory Technicians (MLT), Licensed Practical Nurse (LPN), Doctor of Philosophy (PhD), Doctor of Pharmacy (PharmD), Registered Dietician (RD), Licensed Dietician (LD), Certified Diabetes Educator (CDE), Registered Medical Assistant (RMA), Registered Nurse (RN), and Respiratory Therapist (RT)
[c]SDOH risk is calculated as the ratio of SDOH screener questions with positive screens to the total number of questions answered by patients. Positive screens are indicated by underlined screener responses or otherwise notated with a '*' in eTable 1
[d]Mean and 95% CI are reported for continuous variables
[e]'Other race' includes 'American Indian or Alaska Native, Native Hawaiian or other Pacific Islander and 'Other' as reported in the EHR
[f]'Others' include self-pay, Liability, pending Medicaid and Worker's Compensation

© 2023 Rudisill AC et al. *JAMA Network Open*.

eReferences

1. Hager ER, Quigg AM, Black MM, et al. Development and Validity of a 2-Item Screen to Identify Families at Risk for Food Insecurity. *Pediatrics*. 2010;126(1):e26-e32. doi:10.1542/peds.2009-3146
2. Hall MH, Matthews KA, Kravitz HM, et al. Race and financial strain are independent correlates of sleep in midlife women: the SWAN sleep study. *Sleep*. 2009;32(1):73-82.
3. Health Leads USA. The Health Leads Screening Toolkit. Health Leads. Published 2022. Accessed April 4, 2023. https://healthleadsusa.org/resources/the-health-leads-screening-toolkit/
4. National Association of Communit Health Centers and Partners, National Association of Community Health Centers, Associationof Asian Pacific Community Health Organizations, Association OPC, Institute for Alternative Futures. PRAPARE Toolkit. PRAPARE. Published 2019. Accessed April 4, 2023. https://prapare.org/prapare-toolkit/
5. Elo AL, Leppänen A, Jahkola A. Validity of a single-item measure of stress symptoms. *Scand J Work Environ Health*. 2003;29(6):444-451. doi:10.5271/sjweh.752
6. Manchanda R, Gottlieb L. Upstream Risks Screening Tool and Guide V2.6. HealthBegins. Published 2015. https://www.aamc.org/media/25736/download
7. Sohal H, Eldridge S, Feder G. The sensitivity and specificity of four questions (HARK) to identify intimate partner violence: a diagnostic accuracy study in general practice. *BMC Fam Pract*. 2007;8(1):49. doi:10.1186/1471-2296-8-49
8. Sandel M, Sheward R, Ettinger de Cuba S, et al. Unstable Housing and Caregiver and Child Health in Renter Families. *Pediatrics*. 2018;141(2):e20172199. doi:10.1542/peds.2017-2199
9. Montefiore Health System Office of Community and Population Health. Montefiore Social Determinants of Health Screen.
10. Coleman KJ, Ngor E, Reynolds K, et al. Initial validation of an exercise “vital sign” in electronic medical records. *Med Sci Sports Exerc*. 2012;44(11):2071-2076. doi:10.1249/MSS.0b013e3182630ec1

© 2023 Rudisill AC et al. *JAMA Network Open*.
