# Supplementary material for: Patient and Care Team Perspectives on Social Determinants of Health Screening in Primary Care: A Qualitative Study
Source: JAMA Netw Open. 2023 Nov 28;6(11):e2345444. doi: 10.1001/jamanetworkopen.2023.45444 (PMC10685887; doi:10.1001/jamanetworkopen.2023.45444)
Supplement: Supplement 2. — Data Sharing Statement [file jamanetwopen-e2345444-s002.pdf]

Data Sharing Statement

Rudisill. Patient and Care Team Perspectives on Social Determinants of Health Screening in Primary Care. *JAMA Netw Open*. Published November 28, 2023.
doi:10.1001/jamanetworkopen.2023.45444

Data
Data available: No

Additional Information
Explanation for why data not available: Quantitative data – Such data are available from Prisma Health but restrictions apply to the availability of these data, which were used under agreement for the current study, and so are not publicly available. De-identified data are however available from the authors upon reasonable request and with permission of Prisma Health. Qualitative data - Redacted interview transcriptions are available from the authors upon reasonable request. Any information that might identify a person or the practice in which they work would be removed.
